# Inhibition of glioblastoma malignancy by Lgl1

**DOI:** 10.18632/oncotarget.2580

**Published:** 2014-10-15

**Authors:** Alexander Gont, Jennifer E.L. Hanson, Sylvie J. Lavictoire, Manijeh Daneshmand, Garth Nicholas, John Woulfe, Amin Kassam, Vasco F. Da Silva, Ian A.J. Lorimer

**Affiliations:** ^1^ Centre for Cancer Therapeutics, Ottawa Hospital Research Institute, Ottawa, K1H 8L6, Canada; ^2^ Department of Biochemistry, Microbiology and Immunology, University of Ottawa, Ottawa, Ontario, Canada; ^3^ Department of Pathology and Laboratory Medicine, University of Ottawa, Ottawa, Ontario, Canada; ^4^ Department of Surgery, University of Ottawa, Ottawa, Ontario, Canada; ^5^ Department of Medicine, University of Ottawa, Ottawa, Ontario, Canada; ^6^ Aurora St. Luke's Medical Center, Aurora Health Care, Milwaukee, WI 53215, USA

**Keywords:** Glioblastoma, glioma, Lgl, Lgl1, PTEN, invasion

## Abstract

*lethal giant larvae* (*lgl*) was first identified as a tumor suppressor in *Drosophila*, where its loss repressed the differentiation and promoted the invasion of neuroblasts, the *Drosophila* equivalent of the neural stem cell. Recently we have shown that a human homolog of Lgl, Lgl1 (LLGL1), is constitutively phosphorylated and inactivated in glioblastoma cells; this occurs as a downstream consequence of *PTEN* loss, one of the most frequent genetic events in glioblastoma. Here we have investigated the consequences of this loss of functional Lgl1 in glioblastoma *in vivo*. We used a doxycycline-inducible system to express a non-phosphorylatable, constitutively active version of Lgl1 (Lgl3SA) in either a glioblastoma cell line or primary glioblastoma cells isolated under neural stem cell culture conditions from patients. In both types of cells, expression of Lgl3SA, but not wild type Lgl1, inhibited cell motility *in vitro*. Induction of Lgl3SA in intracerebral xenografts markedly reduced the *in vivo* invasion of primary glioblastoma cells. Lgl3SA expression also induced the differentiation of glioblastoma cells *in vitro* and *in vivo* along the neuronal lineage. Thus the central features of Lgl function as a tumor suppressor in *Drosophila* are conserved in human glioblastoma.

## INTRODUCTION

In *Drosophila*, loss of the *lgl* gene results in overgrowth of both brain and imaginal disc tissue, resulting in death at the late larval stage [1]. Brain overgrowth was shown to be due to overproliferation of neuroblasts, the *Drosophila* equivalent of the neural stem cell. Normal neuroblasts undergo repeated rounds of asymmetric cell divisions to generate a new neuroblast and a daughter cell with limited proliferative capacity that goes on to form the mature cell types (neurons and glia) of the adult fruit fly brain. In *lgl* mutants, instead of these asymmetric divisions, neuroblasts undergo repeated rounds of symmetric cell divisions to generate two neuroblasts [2]. This results in expansion, rather than maintenance, of the neuroblast population, explaining the apparent increase in proliferation described early on for *lgl* mutants. Along with increased proliferation, neuroblasts in *lgl* mutant *Drosophila* spread throughout the larval brain causing abnormalities in brain structure. Transplantation studies showed that brain tissue from *lgl* mutant *Drosophila* was invasive when transplanted into wild type hosts; this invasion was mainly restricted to within the larval brain, with metastases outside the brain being relatively infrequent [3].

Here we are addressing the extent to which the behavior of *Drosophila lgl* mutants is recapitulated in the human adult brain tumor known as glioblastoma. Humans contain two genes with homology to *Drosophila* Lgl; we have focused on Lgl1 (encoded by the *LLGL1* gene) as it is the only homolog that is expressed in mammalian brain tissue [4]. In both *Drosophila* and mammals, Lgl activity is controlled by atypical PKC, which phosphorylates Lgl at its hinge region leading to its inactivation [5, 6]. We have shown previously that Lgl1 is constitutively phosphorylated and inactivated in glioblastoma cells [7]. This inactivation is a downstream consequence of *PTEN* loss, one of the most frequent genetic events in glioblastoma [8, 9]. Currently glioblastoma is an incurable disease with a median survival time of about one year after diagnosis [10]. A key aspect of its malignancy is its highly invasive nature. This invasiveness gives glioblastoma primary tumors their characteristic diffuse borders, and can result in the spread of glioblastoma cells throughout the central nervous system, with frequent involvement of both hemispheres. The pattern of glioblastoma invasion is distinctive, with single cancer cells preferentially traveling along white matter tracts and the outside walls of blood vessels [11]. Another well known aspect of glioblastoma is its phenotypic heterogeneity. Some of this heterogeneity appears to be due to the fact that glioblastoma cells can exist in a range of differentiation states. A subset of cells exists in an undifferentiated neural stem cell-like state; glioblastoma cells in this undifferentiated state are thought to be the key drivers of glioblastoma malignancy [12]. We have previously shown that expression of a non-phosphorylatable, constitutively active version of Lgl1 induces the differentiation of glioblastoma cells from multiple patients along the neuronal lineage in cell culture, a finding that is consistent with the behavior of Lgl in *Drosophila* [7]. Here we have investigated the *in vivo* effects of Lgl1 on glioblastoma malignancy, using a xenograft model that closely mimics the invasive behavior of this disease that is seen in patients.

## RESULTS

### Inhibition of glioblastoma cell motility in cell culture

**Figure 1:**
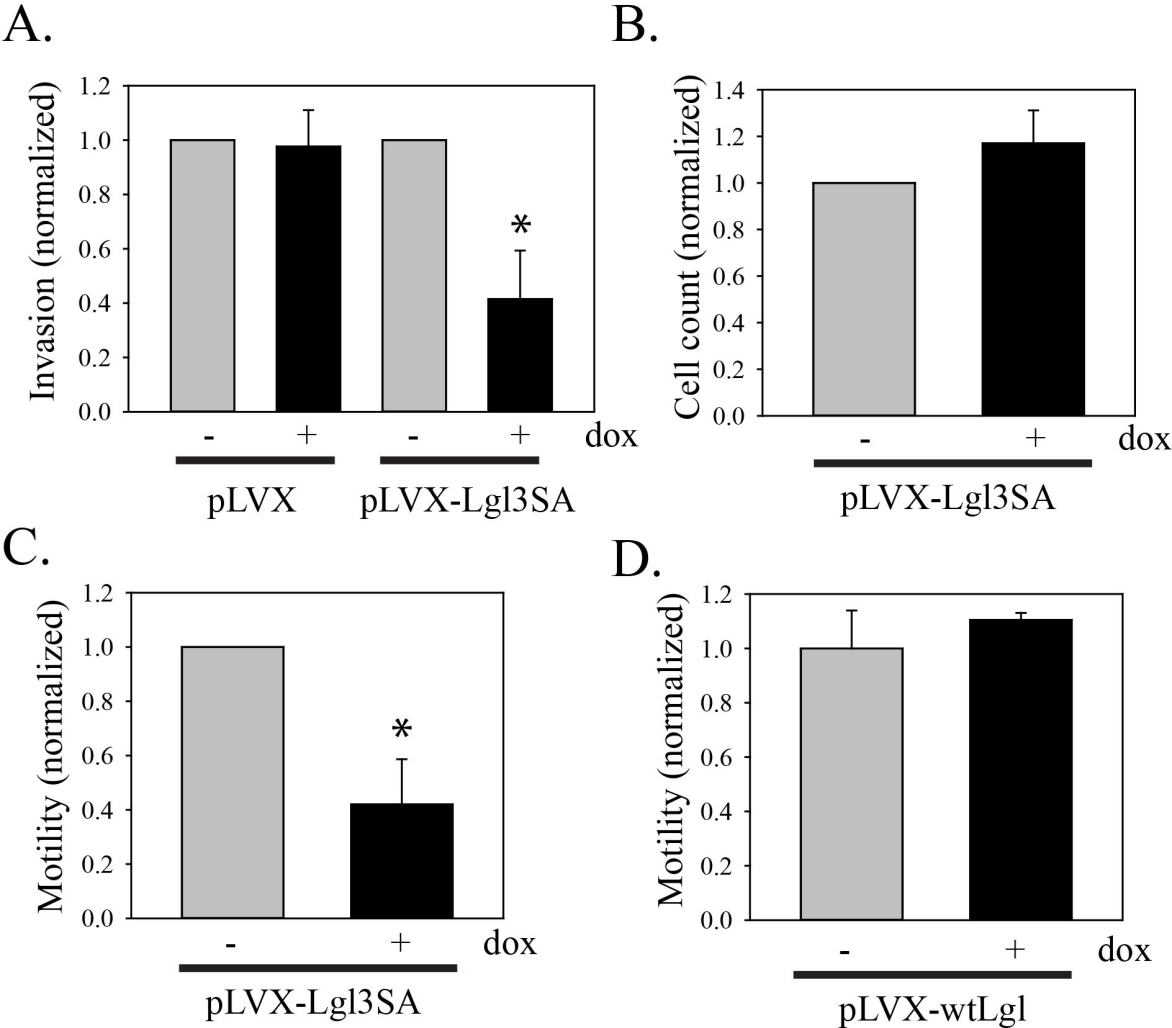
Inhibition of U87MG cell invasion and motility by Lgl3SA **(A)** Invasion of U87MG cells was assessed using Transwell chambers coated with Matrigel. U87MG cells were transduced with Tet activator and either empty vector (pLVX) or vector expressing Lgl3SA (pLVX-Lgl3SA). Cells were then treated with or without doxycycline for three days. Equal numbers of cells were then plated in Transwell plates and 24 h later the number of cells that had crossed the membrane was determined. Data are shown normalized to the untreated doxycycline controls and are the mean of three independent experiments each performed in triplicate. **(B)** Total viable cell counts for U87MG cells with inducible Lgl3SA, with and without treatment with doxycycline for three days. **(C)** To assess motility, U87MG cells transduced with Tet activator and vector expressing Lgl3SA were assayed as in A, except that Transwell membranes without Matrigel were used. Data are shown normalized to the untreated doxycycline controls and are the mean of three independent experiments each performed in triplicate. **(D)** U87MG cells were transduced with Tet activator and vector expressing wild type Lgl1 and assayed as in C. Data shown are from one independent experiment performed in triplicate, normalized to the mean value for untreated cells. Error bars show the mean ± standard deviation. *indicates a *p* value < 0.05.

We first tested the effects of Lgl1 on the invasive properties of the human glioblastoma cell line U87MG. For these experiments, a constitutively active version of Lgl1 was used in which the three hinge region phosphorylation sites were mutated to alanine [13]. This was expressed using a doxycycline-inducible system as described previously [7]. Invasion was evaluated in Transwell membranes coated with Matrigel. As shown in Figure 1A, expression of Lgl3SA caused a 58% reduction in U87MG cell invasion. This effect was not seen in U87MG cells transduced with control vector and treated with doxycycline. Lgl3SA had no effect on total cell numbers under the conditions used here (Figure 1B). To determine if this was an effect of Lgl3SA on the ability of cells to degrade Matrigel, or an effect on motility, we repeated this assay using Transwell membranes without Matrigel. As shown in Figure 1C, Lgl3SA reduced U87MG motility to a similar extent to that seen in the presence of Matrigel, indicating that Lgl3SA is primarily affecting motility. No effect on motility was seen when wild type Lgl was tested for effects on motility, consistent with our previous finding that Lgl is largely inactivated by phosphorylation downstream of PTEN loss in these cells (Figure 1D).

**Figure 2:**
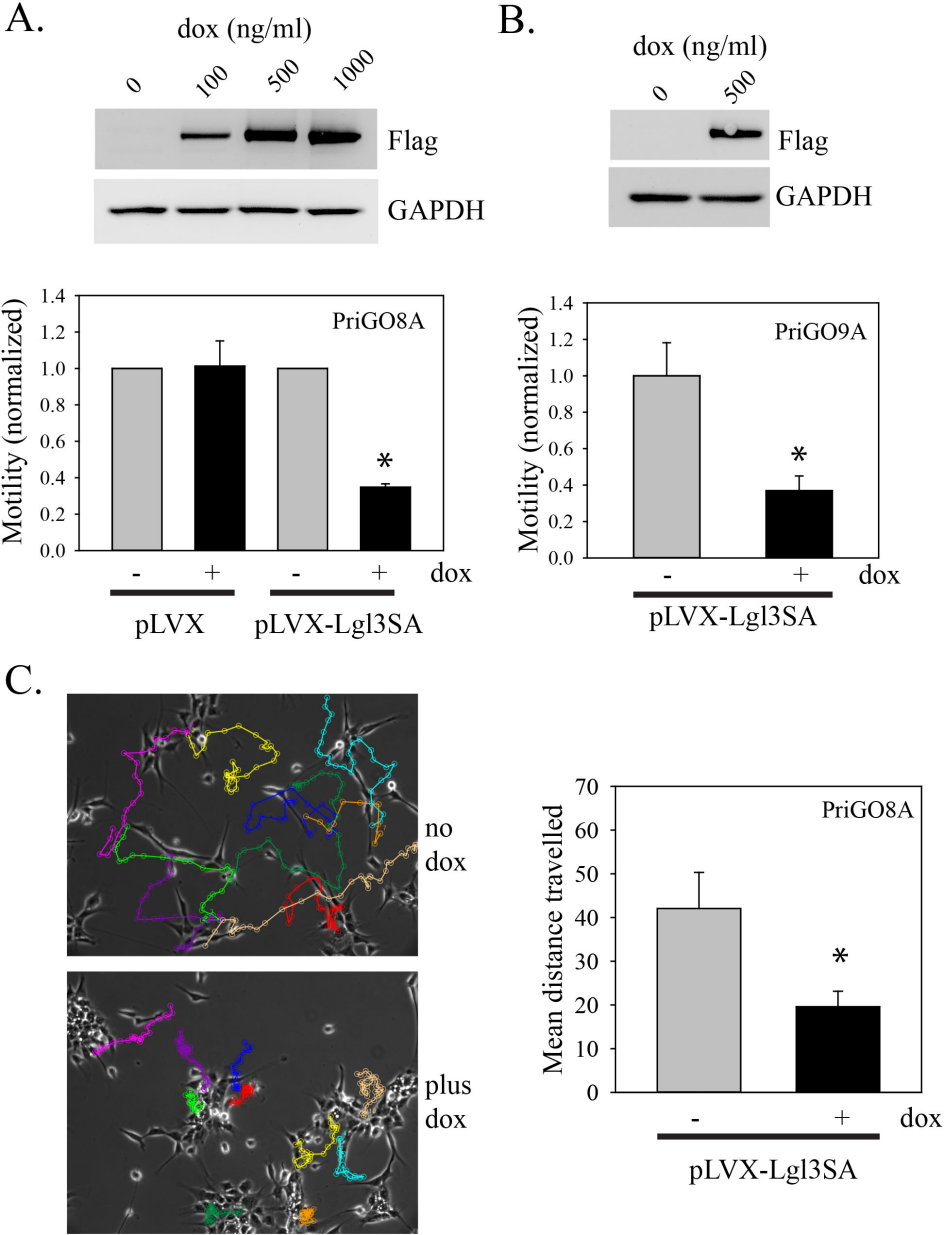
Inhibition of PriGO cell motility by Lgl3SA **(A)** PriGO8A cells were transduced with Tet activator and either empty vector (pLVX) or vector expressing Lgl3SA (pLVX-Lgl3SA). To confirm inducible expression, cells were treated with doxycycline for 24 h and then analyzed by Western blotting for expression of flag-tagged Lgl3SA and GAPDH as a loading control. The bar graph shows the results of motility assays. Cells were treated with or without 500 ng/ml doxycycline for three days. Equal numbers of cells were then plated in Transwell plates and 24 h later the number of cells that had crossed the membrane was determined. Data are shown normalized to the untreated doxycycline controls and are the mean of three independent experiments each performed in triplicate. **(B)** Cells from a second patient (PriGO9A) were transduced with tet activator plasmid and vector expressing Lgl3SA. Confirmation of inducible expression and effects on motility were determined as in A. **(C)** PriGO8A cells transduced with Tet activator and vector expressing Lgl3SA cells were plated in videomicroscopy dishes. After treatment with or without doxycycline for three days, cell movement was recorded by videomicroscopy for 20 h. Tracks of individual cells over 20 h are shown by the colored lines, with each cell assigned a different color. The bar graph shows the mean total distance traveled for ten randomly selected cells under each condition. For all bar graphs in this figure, error bars show the mean ± standard deviation. *indicates a *p* value < 0.05.

**Figure 3:**
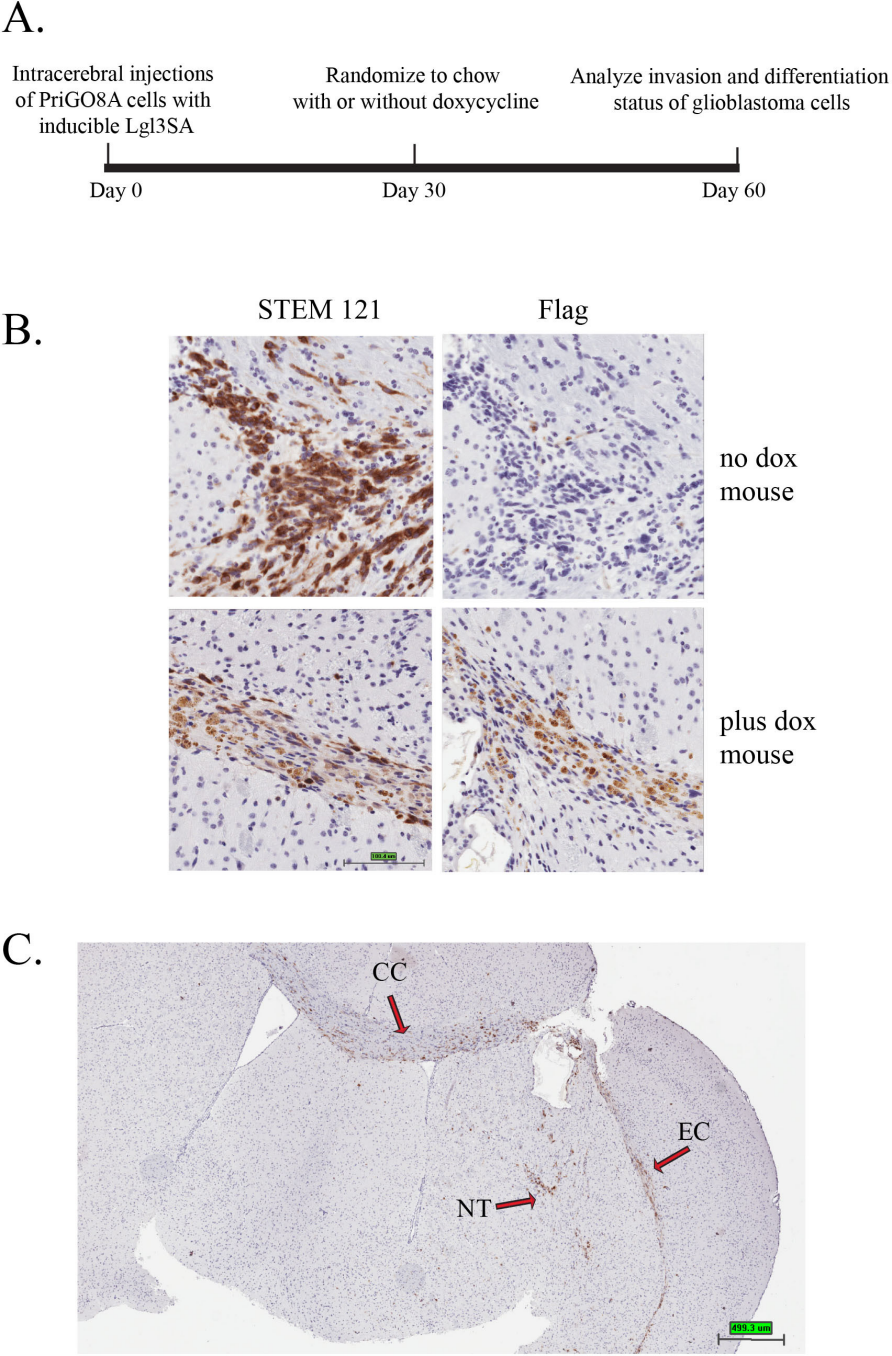
Induction of Lgl3SA *in vivo* **(A)** Experimental design for *in vivo* invasion experiments. **(B)** PriGO8A cells were transduced with lentivirus expressing Tet activator and lentivirus expressing Flag-tagged Lgl3SA under control of the doxycycline-inducible P_tight_ promoter. Cells were then injected intracerebrally in SCID/beige mice. One month after injection of cells, mice were randomized to regular chow or chow with doxycycline. Mice were euthanized one month after randomization. Representative examples of STEM121 and Flag epitope immunohistochemistry performed on serial sections are shown for a mouse that was not treated with doxycycline (top panels) and one that was treated with doxycycline (bottom panels). **(C)** Brain section from a no doxycycline control mouse showing features of invasion in this model. NT, needle track; CC, *corpus callosum*; EC, external capsule.

Although U87MG cells are motile and invasive in cell culture, they are not invasive *in vivo*. We therefore assayed the effects of Lgl3SA on the motility of primary glioblastoma cells isolated from patients under conditions that preserve their invasive properties and that enrich for glioblastoma cells with neural stem cell-like features [7]. We have described the properties of these cells (designated PriGO8A cells) previously [7]; their neural stem cell like characteristics include expression of the neural stem cell markers nestin and sox2, the ability to undergo differentiation along multiple lineages, and the ability to form neurospheres when plated on plastic. In addition these cells form invasive disease in immunocompromised mice; this invasion closely resembles that seen in patients, with single cells invading along white matter tracts including the *corpus callosum* and the external capsule (see Figure 3C). These cells were transduced with lentiviral vectors for doxycycline-inducible expression of Lgl3SA as for U87MG cells (Figure 2A). Testing of these cells for motility in Transwell assays showed that expression of Lgl3SA caused a 65% decrease in their motility (Figure 2A). As seen with U87MG cells, expression of wild type Lgl at similar or higher levels than Lgl3SA had no effect on motility of PriGO8A cells (Figure S1A and B). Similar effects of Lgl3SA on motility were seen in cells isolated from a second patient (Figure 2B). Lgl3SA did not reduce total cell numbers under the conditions used, showing that this effect is not an artifact of effects on proliferation (data not shown). Neither wild type Lgl nor Lgl3SA affected the phosphorylation state of PKCι on Thr555, indicating that they were not suppressing global activation of PKCι (Figure S1C).

As a second method to assess motility that is independent of proliferation, we used time lapse videomicroscopy to monitor motility. PriGO8A cells are highly motile under the culture conditions used (Figure 2C; see also supplementary movies S1 and S2). Tracking of single cells using this method showed that expression of Lgl3SA reduced overall motility to a similar extent to that seen using the Transwell assay technique.

To assess effects of Lgl3SA on glioblastoma invasion *in vivo*, we made use of the doxycycline inducible system to turn on Lgl3SA expression after injection of cells into the cerebrum of immunocompromised mice (Figure 3A). The use of an inducible system avoids potential artifacts due to differences in the ability of genetically modified cells to survive the transplant process into mice; in addition it gives an indication of the potential value of targeting this pathway in established disease. Cells were injected into mice and allowed to establish for a period of one month. Mice were then randomized to regular chow or chow containing doxycycline. One month later, mice brains were analyzed by immunohistochemistry. We first confirmed that Lgl3SA was being expressed in the mice fed chow with doxycycline. To detect injected cells, we used the antibody STEM121, which detects human cells regardless of their differentiation status [14]. Expression of Lgl3SA was detected using antibody to its amino terminal Flag epitope. Figure 3B shows that effective induction of Lgl3SA expression was achieved in mice fed doxycycline chow. STEM121 staining of control mice also showed that cells had invaded extensively in the two month period of the experiment, with invasion along the corpus callosum into the uninjected hemisphere being evident (Figure 3C).

**Figure 4:**
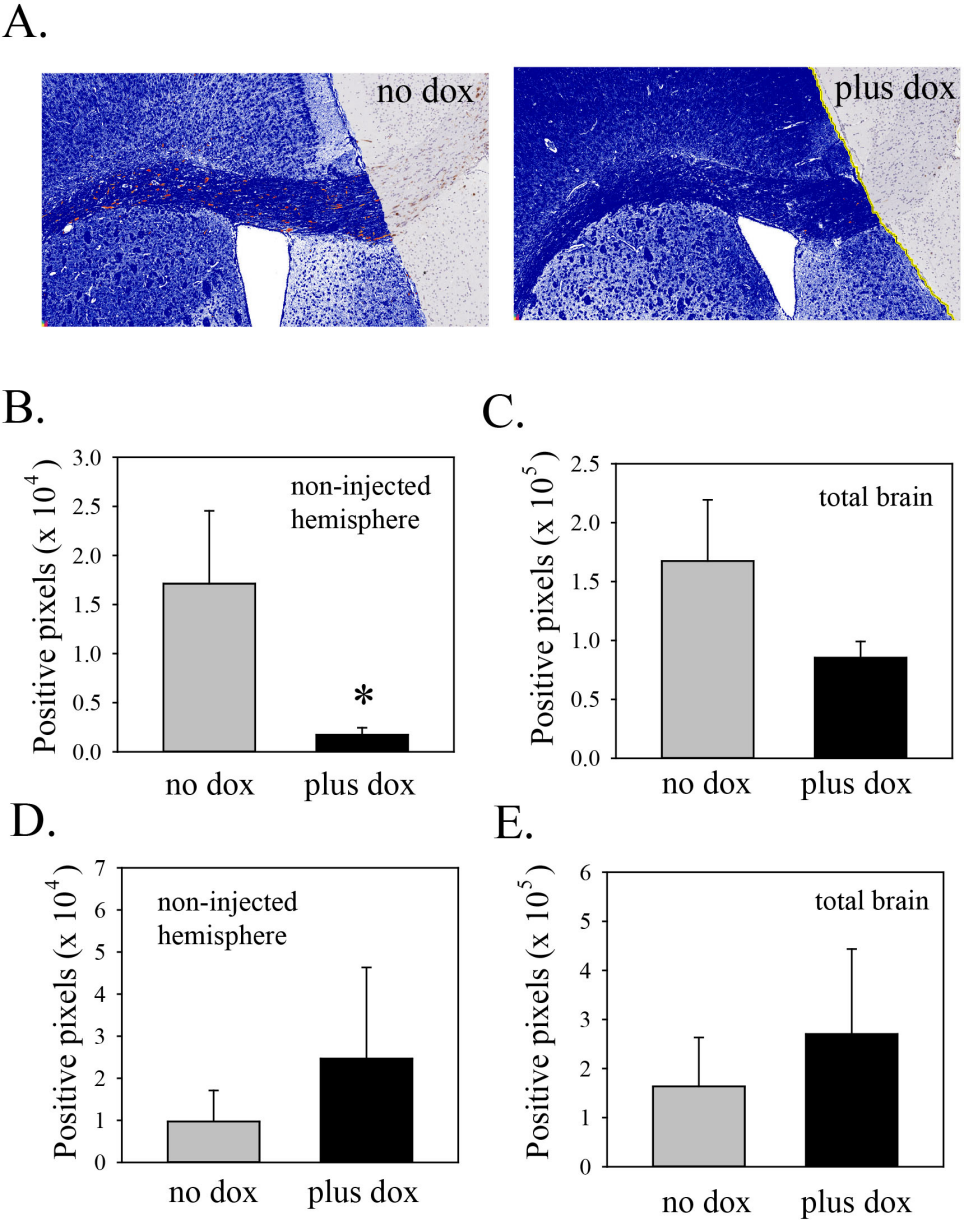
Induction of Lgl3SA reduces invasion *in vivo* **(A)** Representative images from mice on regular chow or doxycycline chow, showing invasion into the non-injected hemisphere, primarily along the *corpus callosum*. The area of analysis is the non-injected hemisphere. Within this area, human cells, identified by STEM121 antibody, are shown pseudocoloured red and negative pixels are pseudocoloured blue. **(B)** Bar graph showing the extent of invasion *(i.e.* positive staining for STEM121 in the uninjected hemisphere). Data are from three mice per group, with four sections analyzed per mouse. **(C)** Bar graph showing total numbers of STEM121 positive pixels in both hemispheres in mice from the regular and doxycycline chow mice. **(D)** As a control, mice were injected with PriGO8A cells transduced with Tet activator lentiviral vector only. One month later mice were randomized to chow with or without doxycycline. After one more month, mice were analyzed for invasion of PriGO8A cells into the non-injected hemisphere as above. Data are from five mice per group, with three sections analyzed per mouse. **(E)** Bar graph showing STEM121 positive pixels in both hemispheres in mice from the regular and doxycycline chow mice from the experiment in D. Error bars show the mean ± standard deviation. *indicates a *p* value < 0.05.

As an objective way to compare the extent of invasion in control and doxycycline-treated mice, total STEM121 positive pixel counts were recorded for the non-injected hemisphere. This was done for four sections from each mouse, taken approximately 20-40 μm apart. An example of this analysis is shown in Figure 4A. Results of the analyses of all the mice are shown in Figure 4B. In mice in which Lgl3SA was induced, there was an 89% reduction in the number of STEM121 positive pixels in the non-injected hemisphere of doxycycline-treated mice and this reduction was statistically significant (Figure 4B). As this could be an indirect effect of a reduction in overall cell numbers, we also recorded total numbers of positive pixels in both hemispheres; these were reduced by 44% (Figure 4C). This difference was not statistically significant and is too small to explain the difference in invasion observed. As a control, we performed a separate experiment using PriGO8A cells transduced with tet activator alone. Mice were injected and randomized to chow with or without doxycycline as above. There was no decrease in invasion in the doxycycline treated animals in this model (Figure 4D and E).

**Figure 5:**
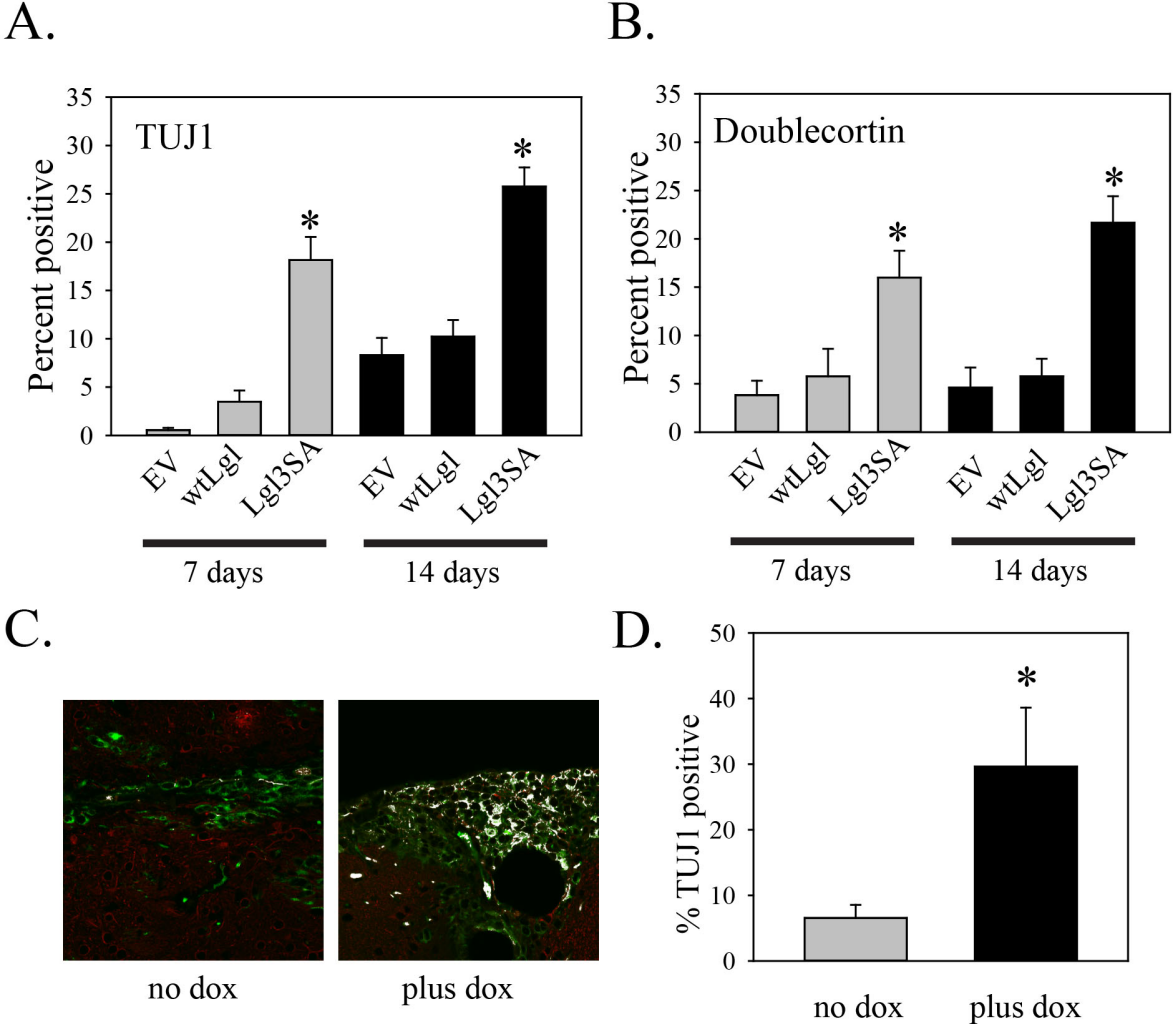
Induction of Lgl3SA promotes differentiation *in vivo*

As we had previously shown that expression of Lgl3SA induced differentiation of glioblastoma cells along the neuronal lineage in cell culture, we also used this experiment to determine if this phenomenon occurred *in vivo*. Our previous work made use of TUJ1 as an early marker of differentiation along the neuronal lineage. Analysis of an additional early neurogenesis marker, doublecortin [15], in cell culture supported this finding (Figure 5A and B). The rise in markers of neuronal differentiation was only seen in cells transduced with Lgl3SA, not wild type Lgl, again consistent with the idea that Lgl is inactivated by phosphorylation in these cells (Figure 5A and B). The increase in TUJ1 was seen after seven and fourteen days of Lgl3SA induction, but not after one day of induction (Figure S1D). To assess differentiation *in vivo*, sections from the mouse experiment were analyzed by double immunofluorescent immunohistochemistry for STEM121 and TUJ1. Examples of this are shown in Figure 5C and in Figure S2. Figure 5D shows the data for all mice, with three different sections analyzed per mouse. Induction of Lgl3SA resulted in a 4.5 fold increase in the numbers of pixels that were double positive for STEM121 and TUJ1, showing that induction of Lgl3SA promotes differentiation along the neuronal lineage *in vivo*.

**(A and B)** Analysis of TUJ1 (A) and doublecortin (B) expression in PriGO8A cells transduced with control empty lentiviral vector (EV), or lentiviral vectors expressing wild-type Lgl or Lgl3SA. Immunofluorescence for TUJ1 and doublecortin was performed on the indicated number of days after transduction and the percent of positive cells was counted. Data are the mean ± standard error from five randomly selected fields per condition. **(C)** Immunohistochemistry for STEM121/TUJ1 double positive cells. Each section was stained with STEM121 antibody (green: identifies human cells) and TUJ1 (red: identifies cells differentiated along the neuronal lineage in both human and mouse tissue). White areas show cells that are both STEM121 and TUJ1 positive. Representative images from one untreated and one doxycycline-treated mouse are shown. Larger versions of these images, along with additional images, are shown in Figure S2. **(D)** Bar graph showing the percent of STEM121 positive pixels that are also TUJ1 positive in mice under the two conditions. Data are from three mice per group, with three sections analyzed per mouse. Error bars show the mean ± standard deviation. *indicates a *p* value < 0.05.

## DISCUSSION

*Drosophila lgl* mutants showed two key changes with respect to neuroblast behavior: first, the neuroblast population expands as a consequence of its inability to differentiate; second the neuroblasts disseminate throughout the larval brain, while showing limited ability to metastasize to other sites. We show here that Lgl inactivation in human glioblastoma has similar consequences. A key difference between *Drosophila lgl* mutants and human glioblastoma is the mechanism of Lgl inactivation. In human glioblastoma, Lgl1 is not inactivated by mutation; instead it is inactivated by constitutive phosphorylation that is a consequence of *PTEN* loss. Consistent with this, we found that the effects of Lgl1 on both invasion and differentiation were only seen when a non-phosphorylatable, constitutively active version of Lgl1 was used. As partial or complete loss of *PTEN* occurs in 85% of glioblastomas, and *PTEN* loss is thought to be an early genetic event, this would appear to be a key pathway in gliomagenesis. The Strand laboratory has previously described a mechanism for Lgl inactivation in human cancer (primarily breast cancer) in which Lgl expression is repressed by the transcription factor Snail as an essential step in the epithelial to mesenchymal transition [16]. This effect was restricted to Lgl2, with Snail having no effect on Lgl1 expression; as Lgl2 expression is absent in mammalian brain this pathway is unlikely to play a role in glioblastoma [4]. In theory, the two pathways could act in concert in some cancer types to repress both Lgl1 and Lgl2 function.

As we see effects on both differentiation and invasion, one question is whether the effects on invasion are a consequence of differentiation. This interpretation is suggested by the well documented ability of neural stem cells to migrate through the brain in response to injury [17]. In addition, it has been shown that glioblastoma cells treated with serum express differentiation markers and lose their invasive properties [18]. However our data suggest that Lgl1 primarily has a direct effect on glioblastoma cell motility that is independent of its effects on differentiation, because it is also seen in U87MG cells, which do not have the differentiation potential of the primary glioblastoma cells. This effect on motility may reflect a need to inactivate Lgl1 to allow the dynamic changes in the cell polarity required for coordinated cell movement [19]. Biochemically, the repression of cell motility by Lgl1 may be mediated in part by its effects on cytoskeletal non-muscle myosin IIA [20–22]. Although the effects of Lgl3SA appear to be primarily a direct consequence of the inhibition of motility, it is possible that differentiation contributes further to reduced invasion *in vivo*. This possibility is suggested by the fact that Lgl3SA effects on invasion *in vivo* (assessed after one month of Lgl3SA induction) are larger than those seen *in vitro*. We also see a trend towards a reduction in total cell numbers *in vivo*. As this is not seen in short term *in vitro* experiments, this may also be a long term consequence of differentiation, with a concomitant reduction in proliferative potential. A current model depicts cancer cells as stochastically transitioning between differentiation states to reach a phenotypic equilibrium (including transitions from non stem-like to stem-like) [23]. In the context of this model, Lgl inactivation in glioblastoma would shift this equilibrium so that on average a greater number of cells occupy a less differentiated state. Further studies are required to determine whether this affects response to radiation and temozolomide therapy.

In summary, our data support a novel mechanism for gliomagenesis, in which loss of *PTEN* leads to inactivation of a second tumor suppressor, Lgl1, which in turn promotes invasion while repressing differentiation. Lgl1 inactivation is due to phosphorylation by PKCι, which is activated by *PTEN* loss. Our findings, along with recent data showing a lack of toxicity for neuron-specific PKCι knockouts in mice [24], support further study of PKCι as a therapeutic target for glioblastoma.

## METHODS

### Antibodies

The following antibodies were used: FlagM2 mouse monoclonal (Sigma-Aldrich, Oakville, ON, Canada); Stem121 mouse monoclonal (Stemcells Inc., Newark, CA, USA); TUJ1 rabbit monoclonal antibody (Covance, Princeton, NJ, USA); doublecortin rabbit polyclonal antibody (Cell Signaling Technology, Danvers, MA, USA). PKCι mouse monoclonal antibody was from BD Transduction Laboratories (Mississauga, ON, Canada) and anti-Phospho [Thr555]-PKCι rabbit polyclonal antibody was from Invitrogen (Carlsbad, CA, USA).

### Cell culture

U87MG cells were grown in Dulbecco's Modified Eagle medium supplemented with 100 units/ml penicillin, 100 μg/ml streptomycin and 10% fetal bovine serum at 37°C and 5% CO_2_. PriGO cells were described previously [7] and were grown on laminin-coated plates in Neurobasal A medium supplemented with B27, N2, EGF and FGF2 at 37°C in 5% O_2_/CO_2_. PriGO cells were used at passage < 20 to avoid potential loss of multipotency [25]. Cells with inducible transgene expression were generated by transduction with lentiviral vector expressing Tet activator (Clontech, Mountain View, CA, USA) and selection in G418, followed by transduction with inducible lentiviral vectors made from pLVX-Tight-Puro (Clontech, Mountain View, CA, USA) and selection in puromycin, as described previously [7].

### *In vitro* invasion and motility assays

U87MG or PriGO cells transduced with a doxycycline inducible empty vector, wildtype Lgl or LGL-3SA cDNA were incubated with or without doxycycline for three days (100 ng/mL for U87MG and 500 ng/mL for PriGO8A). Cells were then counted and re-plated in the top compartment of 8 μm Transwell inserts with or without Matrigel (Corning BioCoat, Corning, NY, USA) and in parallel in a 24 well plate. For U87MG experiments 10% serum containing media was added to the bottom compartment. For PriGO cells laminin was added directly to the media in both the top and bottom compartments. Doxycycline was added to both compartments at the corresponding doses. After 22–24 hour incubation, cells remaining in the top compartment were scraped off with a swab. Cells were fixed and stained using the Kwik Diff staining kit (ThermoElectron, Pittsburgh, PA, USA). Migrated cells were counted within 5 random fields at 40x magnification of 3 separate replicates and counts were normalized to the average cell count of each experimental condition from the parallel 24 well plate.

### Videomicroscopy

PriGO8A cells transduced with doxycycline inducible Lgl3SA were plated on laminin coated Bioptechs delta-T dishes (Butler, PA, USA) in 1mL media. Cells were grown for three days at 37°C in 5% O_2_/CO_2_ in the presence or absence of 500 ng/mL doxycycline in the media. Cells were maintained at 37°C in a sealed chamber for the duration of the image acquisition. Phase contrast images of the cells were taken at 5 min intervals for 17 h using the through 10x objective of the ZiessAxiovert 200 M microscope equipped with a AxioCamHRm CCD camera(Zeiss, Göttingen, Germany). Motility was quantified as the average distance per point every five frames of ten cells per condition using the MtrackJ plugin [26] in ImageJ software (National Institutes of Health, Bethesda, Maryland, USA).

### Mouse model

Experiments were carried out in accordance with the recommendations of the Animal Care Committee at the University of Ottawa. PriGO8A cells were transduced with a doxycycline inducible lentiviral vector for Lgl3SA cDNA expression. 1 × 10^5^ cells in 10 μL sterile PBS were injected intrastriatally (right side of the skull approximately 0.5mm above the coronal suture and 2mm from the sagittal suture) into 4-6 week old Fox Chase SCID/beige mice (Charles River Laboratories, Wilmington. MA). Mice were kept under normal conditions for 4 weeks after which mice were randomized into two groups. One group was fed regular chow as control and one group was fed nutrient matched doxycycline containing chow (Rodent Diet #2018 - 625 Doxycycline, Harlan, IN, USA).

### Immunohistochemistry

Whole brains were harvested, and fixed in formalin for 48h. Brains were cut in the coronal plane at the site of injection and paraffin embedded. Antigen retrieval on 5 μm sections was performed in citrate buffer (Vector, Burlingame, CA, USA) in a decloaking chamber (Biocare Medical, CA, USA). For colorimetric immunohistochemistry the DakoEnVision+ system HRP labeled polymer was used (Dako North America, Carpinteria, CA, USA) and sections were developed using DAB Peroxidase Substrate Kit (Vector, Burlingame, CA, USA) and counterstained with haematoxylin (Vector; Burlingame, CA, USA). For fluorescent immunohistochemistry double labeling experiments Alexa-flour conjugated secondary 488 and 555 antibodies were used (Life Technologies, Eugene, OR, USA). Colorimetric immunohistochemistry slides were scanned using the ScanScope CS2 (Aperio, CA, USA). Fluorescent immunohistochemistry images were acquired under 40x magnification using the Zeiss Observer Z1 microscope connected to a Zeiss LSM 510 Meta confocal unit (Zeiss, Göttingen, Germany). Positive pixel counts of immunohistochemistry slides were performed using Aperio ImageScope software (Version 11.2.0.780, Aperio, CA, USA). Fluorescent IHC double positive pixel counts of TUJ1:Stem121 were quantified using Zen 2008 software (Zeiss, Germany) Double positive pixels were highlighted and pseudocolored white using the “co-localization finder” plugin in ImageJ software (http:rsb.info.nih.gov/ij/plugins/colocalization-finder.html).

### Statistical analyses

All statistical analyses were performed using SigmaPlot12 software. Comparisons between two groups were performed using two-tailed t-tests with a p value <0.05 considered significant.

## SUPPLEMENTARY FIGURES AND MOVIES


