# Supplementary material for: Inhibition of glioblastoma malignancy by Lgl1
Source: Oncotarget. 2014 Oct 15;5(22):11541–51. doi: 10.18632/oncotarget.2580 (PMC4294391; doi:10.18632/oncotarget.2580)
Supplement: Supplementary file 1 [file oncotarget-05-11541-s001.pdf]

SUPPLEMENTARY FIGURES

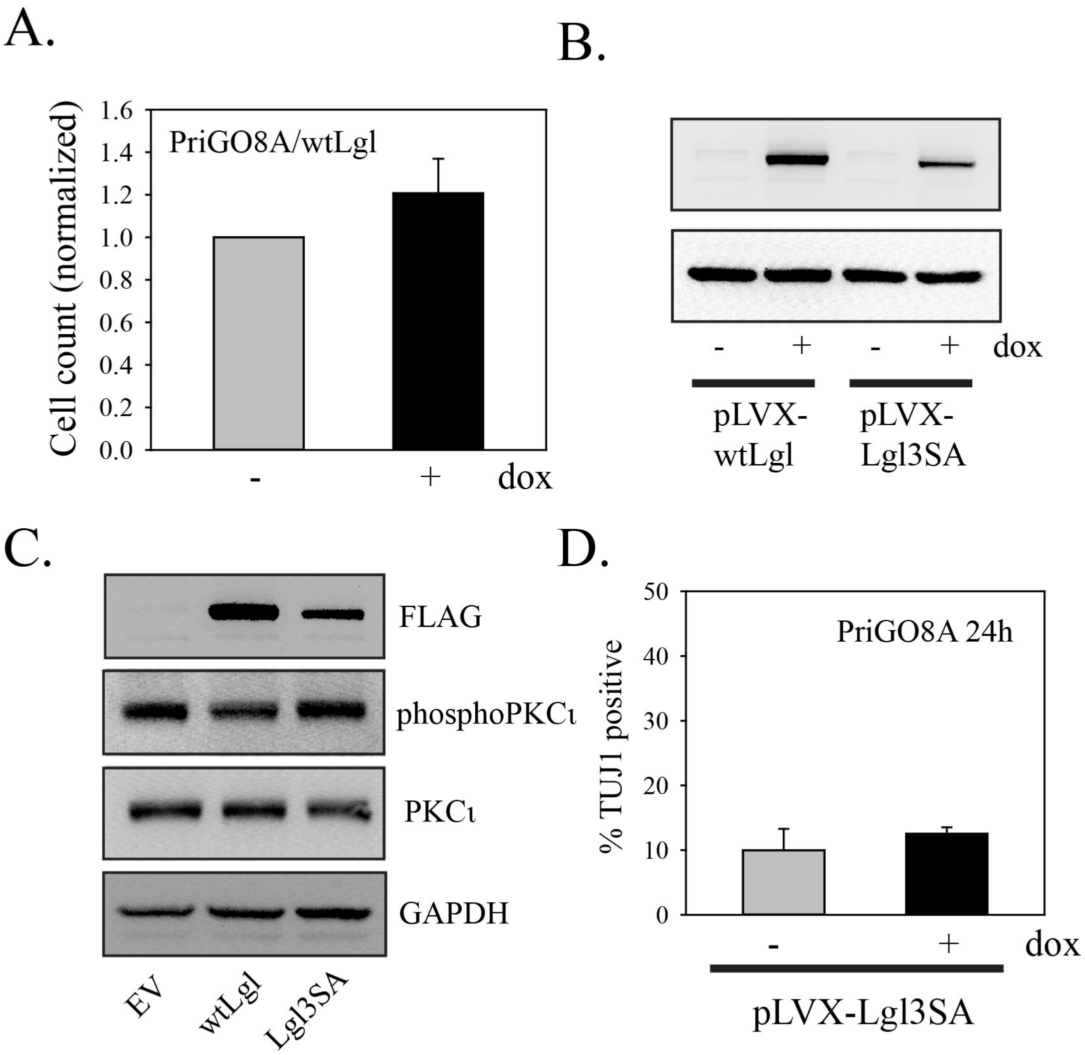

Supplementary Figure S1: (A) PriGO8A cells were transduced with Tet activator and vector expressing wild type Lgl (pLVX-wtLgl). Cells were treated with or without 500 ng/ml doxycycline for three days. Equal numbers of cells were then plated in Transwell plates and 24 h later the number of cells that had crossed the membrane was determined. Data are shown normalized to the untreated doxycycline controls and are the mean of three independent experiments each performed in triplicate. **(B)** Comparison of inducible expression of wild type Lgl and Lgl3SA in PriGO8A cells. PriGO8A cells were transduced with Tet activator and either vector expressing wild type Lgl (pLVX-wtLgl or vector expressing Lgl3SA (pLVX-Lgl3SA). To confirm inducible expression, cells were treated with doxycycline for 48 h and then analyzed by Western blotting for expression of flag-tagged Lgl and Lgl3SA. GAPDH was used as a loading control. **(C)** Effects of Lgl expression on PKCι activation. PriGO8A cells were transduced with lentiviral vectors expressing either wild type Lgl or Lgl3SA. 24 h later cells were analyzed for effects on PKCι activation, as assessed by Western blotting with antibody to PKCι phosphorylated on Thr555 as well as total PKCι levels. **(D)** Analysis of TUJ1 expression in PriGO8A cells transduced with Tet activator and vector expressing Lgl3SA after 24 hours of doxycyline treatment. Immunofluorescence for TUJ1 was performed and the percent of positive cells was determined. Data are the mean ± standard error from five randomly selected fields per condition.

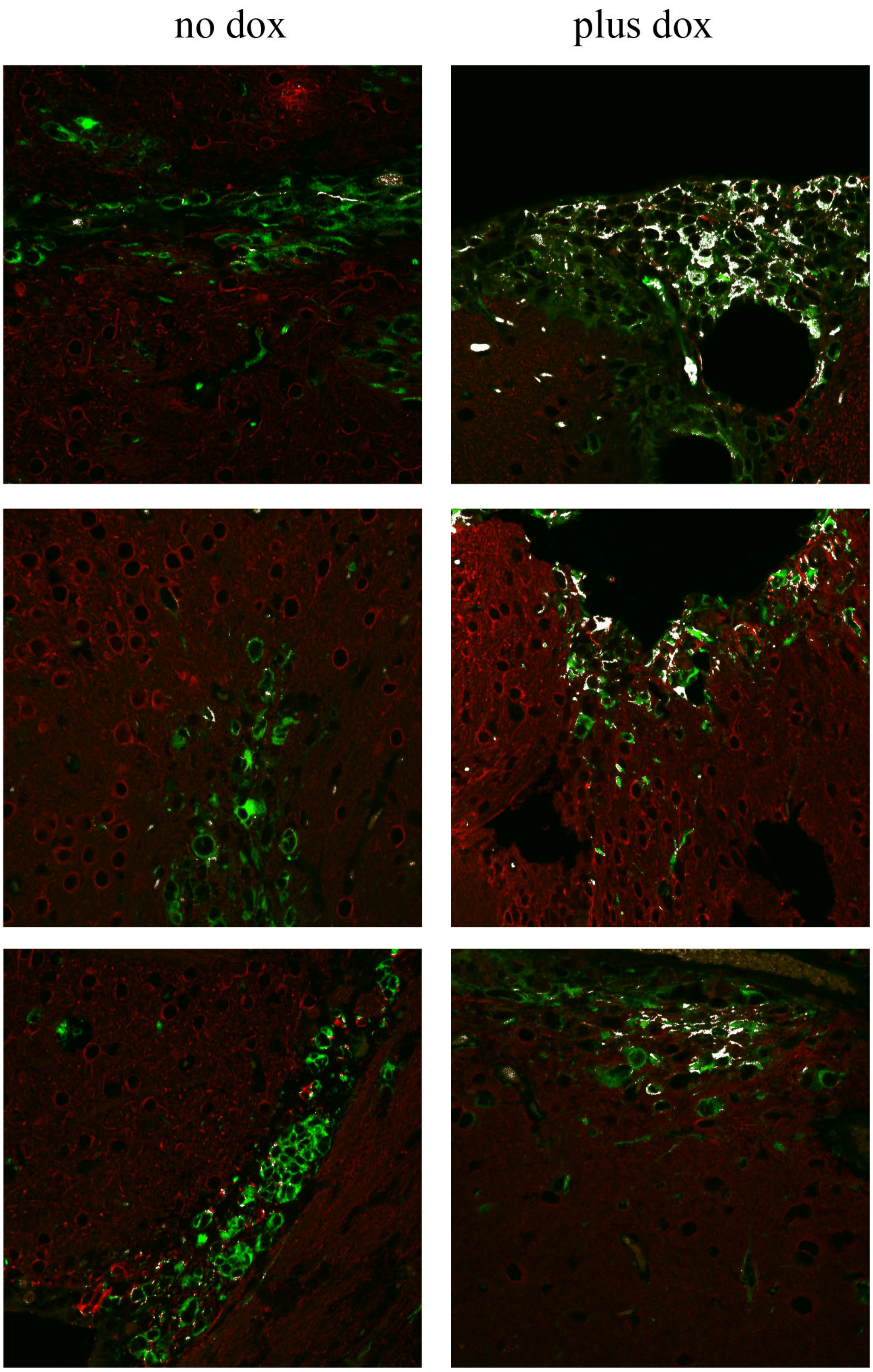

Supplementary Figure S2: The top two panels are enlarged versions of the images shown in Figure 5C. Four additional images, each from a different mouse (two on regular chow and two on doxycycline-containing chow), are also shown. Details of the analysis performed are described in Figure 5C.

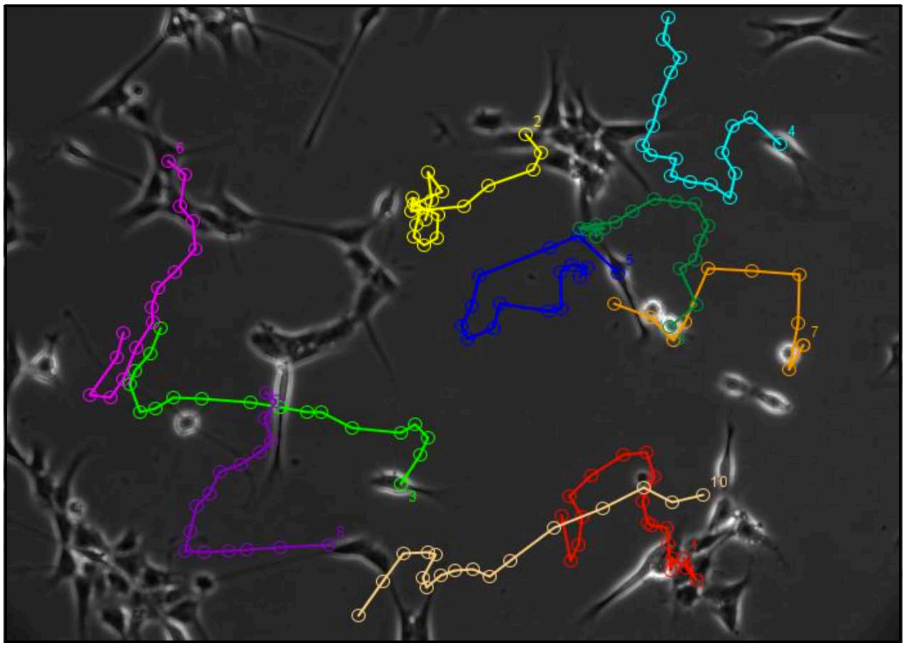

Supplementary movie S1: Videomicroscopy of PriGO8A cells transduced with doxycycline inducible Lgl3SA in the absence of doxycycline.

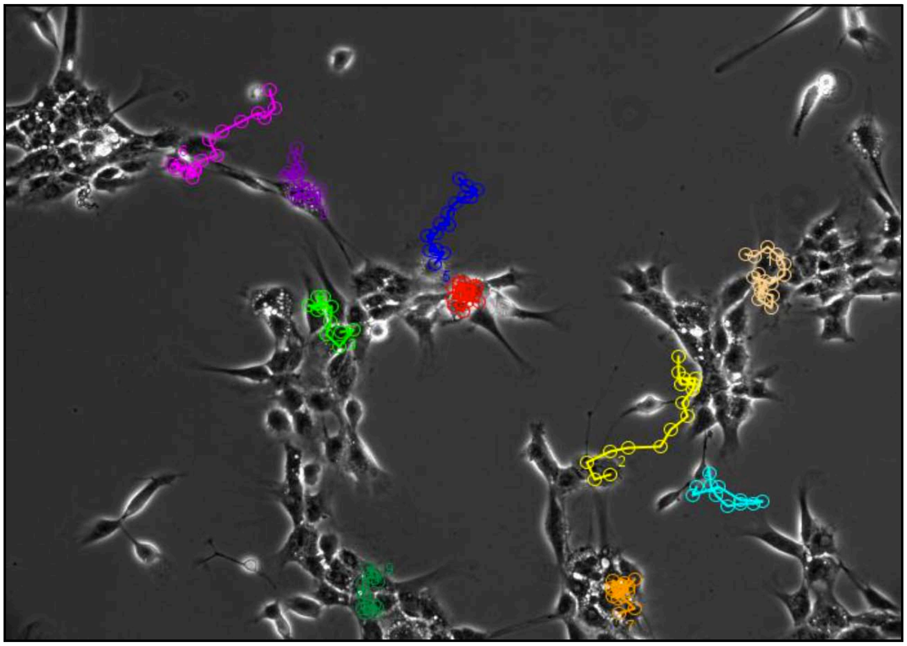

Supplementary movie S2: Videomicroscopy of PriGO8A cells transduced with doxycycline inducible Lgl3SA in the presence of doxycycline.
